# Polymorphism of *pfmdr1* Gene Mutation Conferring Resistance to Artemisinin‐Based Combination Therapy in *Plasmodium falciparum* in Patients at Gulu Regional Referral Hospital in Northern Uganda

**DOI:** 10.1155/japr/8966853

**Published:** 2026-06-09

**Authors:** Florence Peace Amito, Harriet Angwech, Lonzy Ojok, Godfrey Wokorach, Stephen Ochaya, Quinto Ogwang, Robert Opiro, Richard Echodu

**Affiliations:** ^1^ Department of Biology, Faculty of Science, Gulu University, Gulu City, Uganda, gu.ac.ug; ^2^ Department of Pathology, Faculty of Medicine, Gulu University, Gulu City, Uganda, gu.ac.ug; ^3^ Gulu University Multifunctional Research Laboratories, Gulu City, Uganda; ^4^ Department of Biology, Faculty of Science, Muni University, Arua City, Uganda, muni.ac.ug; ^5^ Department of Clinical Pathology, Uppsala Academic Hospital, Uppsala, Sweden; ^6^ Gulu Regional Referral Hospital, Gulu City, Uganda

**Keywords:** antimalarial resistance, *Kelch 13* gene, parasite load, *Plasmodium falciparum*, single-nucleotide polymorphism

## Abstract

**Background:**

Malaria is one of the most devastating infectious diseases in humans, and antimalarial drugs have been used to combat it with minimal success. Worldwide, malaria treatment is threatened by the emergence and spread of artemisinin resistance, which is associated with mutations in the *PfK13* propeller domain. In Sub‐saharan Africa, data relating to the prevalence of *Plasmodium falciparum* malaria infection in association with the *Kelch 13* mutations are mainly from research settings outside disease‐endemic areas. This study is aimed at establishing the prevalence of *P. falciparum* malaria infection in association with *Kelch 13* mutations among patients presenting with fever at Gulu Regional Referral Hospital (GRRH) in northern Uganda.

**Methods:**

This cross‐sectional study enrolled all participants presenting with fever at GRRH between April 2022 and January 2024. Data on adults and children aged ≥ 6 months with fever and confirmed diagnosis of malaria using mRDT, microscopy, and PCR were collected. Parasite DNA was extracted using the Chelex method and sequenced for multidrug resistance genes, and Sanger customized CRF forms were used to capture variables on social demographics, clinical presentation, and treatment. Data were analyzed using IBM SPSS Version 25, and the sequenced data were analyzed using molecular evolutionary genetic analysis (MEGA) Version 11.1.10. All sequences from a single population were aligned using the National Center for Biotechnology Information (NCBI) database.

**Results:**

In total, 353 participants were recruited, and the overall prevalence of *P. falciparum* malaria was 60.6% (*n* = 214), with the highest number of cases registered in Gulu City (24.9%). Women were the most affected participants (37.1%). The most common clinical presentations among the participants were fever (91.8%; *n* = 324), chills (90.7%; *n* = 320), and headaches (72.0%; *n* = 254). Genotyping results of the mutant genes showed that of all 214 *P. falciparum* isolates examined, the *pfmdr1* SNP at Codon 1034 1042 (29.6%, *n* = 94) had the highest prevalence, followed by the *pfmdr* SNP at Codon 86 184 (28%, *n* = 89), and the SNP fragment at codon 1246 (25.8%, *n* = 82) recorded the lowest prevalence. *Kelch 13* propeller gene, known to be associated with artemisinin resistance, was also isolated in 16.7% (*n* = 53) of the samples. There was a 90.1% (*n* = 318) prevalence of the SNPs 86 184, 1034 1042 of the *pfmdr1* gene, and *K13* propeller gene, with no significant difference between the sexes (*p* = 0.756). The SNP at Codon 1246 of *pfmdr1* showed a significant difference between the location and mutation (*p* = 0.017). The median parasite load in patients with mutations in 86 184, 1034 1042, and *K13* propeller genes varied significantly among patients who received treatment *p* ≤ 0.0001, *p* = 0.0061, and *p* = 0.012, respectively.

**Conclusion:**

The presence of *pfmdr1* mutant genes suggests resistance of *P. falciparum* to most antimalarial drugs used in treatment. Therefore, it is important to monitor the prevalence of *Kelch 13* mutations and *P. falciparum* to contribute to global efforts to control and eliminate malaria.

## 1. Introduction

Undoubtedly, morbidity and mortality due to *Plasmodium falciparum* malaria remain high. Sub‐saharan Africa (SSA) contributes 234 million malaria cases, accounting for 95% of all malaria cases globally in 2021, with Uganda accounting for 5% [1]. There were 232 million cases in 2019, 245 million in 2020, and 247 million in 2021 [2]. In Uganda, malaria remains the leading cause of death, especially in children under 5 years of age, with over 90% of the population at risk, making Uganda one of the countries with the highest global burden of malaria cases [2]. Globally, *P. falciparum* is the most frequent cause of severe malaria associated with poor outcomes [1].

Artemisinin‐based combination therapies (ACTs) have greatly contributed to the global decline in illness and death from malaria [3]. However, the novel emanation of artemisinin resistance in Eastern Africa threatens the efficacy of breakthrough treatments [4]. *Plasmodium falciparum Kelch 13 (pfk13*) gene mutations confer artemisinin resistance; however, only a few are validated markers of resistance, defined by both in vitro resistance and delayed parasite clearance in treated patients [3]. Mutations in *pfk13* (*Kelch 13* [*K13*]) were definitively associated with artemisinin resistance [5]. Despite the gradual emergence of artemisinin resistance in other parts of the world, a strong correlation between artemisinin resistance and*K13* mutations has not been observed. Numerous mutational markers associated with artemisinin resistance have been identified in in vitro drug adaptation studies [6].

The high prevalence of wild‐type alleles N86 and D1246 in *P. falciparum* isolates could translate to decreased sensitivity to artemether–lumefantrine [7]. A definitive study in Gulu at St. Mary′s Hospital Lacor in Northern Uganda showed that in vivo resistance increased by 2% between 2017 and 2019, mutation prevalence increased by 15.9% between 2015 and 2019, and the pervasiveness of parasites with *K13* mutations increased primarily because of the increase in regularity of the A675V and C469Y alleles [4]. The data on the prevalence of *P. falciparum* malaria infection in association with *K13* mutation among patients presenting with fever at Gulu Regional Referral Hospital will enhance the establishment of the prevalence of *P. falciparum*, particularly in association with the *K13* gene. This is crucial because *K13* mutations are associated with resistance to ACTs, which are frontline drugs against malaria [8]. Monitoring the prevalence of *P. falciparum* in association with *K13* mutation genes will help to track their emergence and spread, thereby guiding treatment policies and assessing the efficacy of the ACTs in specific regions. This prompts the need for alternative treatments and prioritization of interventions, thereby contributing to the global effort to control and eliminate malaria. This information will guide further studies aimed at understanding the mechanisms of drug resistance and developing new antimalarial drugs and alternative treatment strategies [7]. This study is aimed at establishing the prevalence of *P. falciparum* malaria infection associated with *K13* mutations in patients presenting with fever at GRRH.

## 2. Materials and Methods

### 2.1. Study Design and Settings

This study was conducted on adults and children aged 6 months or older who visited the GRRH hospital between April 2022 and January 2024. The hospital is located within Gulu City, 356 km north of Uganda′s capital Kampala. The hospital has 370 beds and offers outpatient and laboratory services. Most of the severe malaria cases and complications are treated at this hospital.

### 2.2. Sample Size

The sample size was calculated using the formula of Daniel [9] that is *n* = (*Z* ^2^ *P*(1 − *P*))/*d* ^2^, where the parameters were *Z* = 1.96 (for a 0.05 level of significance), expected period prevalence or proportion was considered to be *P* = 0.3, and the precision *d* = 0.05 was used to produce good precision and smaller error of estimate. The calculated sample size was 323, and 10% of nonrespondents were added, resulting in a total sample size of 355.

#### 2.2.1. Inclusion and Exclusion Criteria

All adults and children aged ≥ 6 months with a confirmed diagnosis of malaria were included if they met any one or more of the Uganda Clinical Guidelines 2016: National Guidelines for the Management of Common Conditions of the Republic of Uganda Ministry of Health in December 2016 [10]. These criteria included severe anemia, prostration (generalized weakness such that the person is unable to sit, stand, or walk without assistance), shock (compensated or decompensated), multiple convulsions (two or more convulsions in 24 h), impaired consciousness, pulmonary edema, respiratory distress, hemoglobinuria, hypoglycemia, and renal failure. Participants who presented with diagnoses other than malaria were excluded from this study.

#### 2.2.2. Sample Collection

All eligible participants who presented with fever at the OPD clinic GRRH were briefed on the test before obtaining written consent. Participants under 18 years of age were allowed to provide consent through their parents or legal guardians (assent). About 4 mL of blood from each enrolled participant was collected in EDTA vacutainers, and approximately five drops were made on FTA cards for the dry blood spots, which were then allowed to dry and kept in individual plastic bags with desiccant and stored at room temperature. Malaria diagnosis in this facility was performed using blood smears and rapid diagnostic test kits, both of which can identify *P. falciparum* infection. Malaria diagnosis was performed on the sample in the EDTA vacutainer using multispecies mRDT by Biotrol Laboratories Private Limited, New Delhi, India (Biotrol‐An ISO 1345‐2016 Certified Company), and malaria microscopy was performed using thick and thin smears stained with 10% Giemsa made from Giemsa powder manufactured by Loba Chemie Private Limited, Mumbai, India.

#### 2.2.3. DNA Extraction

DNA was extracted from the dry blood spot on the FTA cards using the Chelex‐100 (Bio‐Rad Laboratories, California, United States) method.

#### 2.2.4. PCR for *Plasmodium* Detection

Nested PCR (nPCR) was adopted from [11] and Snounou et al. [12] using the primer sets PLUf 5 ^′^‐ TTA AAA TTG TTG CAG TTA AAA CG 3 ^′^ and PLUr 5 ^′^ ‐ CCT GTT GTT GCC TTA AAC TTC‐3 ^′^. The PCR master mix was prepared in a total volume of 25 *μ*L containing 12.5‐*μ*L 2x Taq polymerase PCR ready mix, 0.5 *μ*L of 100 *μ*M each of the primers, 9.5 *μ*L of nuclease‐free water and 2 *μ*L of extracted DNA. The PCR was performed under the following conditions: initial denaturation at 94^o^C for 1 min; 35 PCR cycles of denaturation at 94^o^C for 1 min, annealing at 58^o^C for 2 min, and elongation at 72^o^C for 5 min; and a final hold at 4^o^C infinitely. To ensure the validity of the test, a known *Plasmodium*‐positive sample and a negative control sample without the DNA template were used in all reactions as positive and negative controls, respectively.

#### 2.2.5. PCR for *Plasmodium* Species Detection

The amplified PCR product was used as a template for the second PCR for the identification of the species, *P. falciparum:* FALf 5 ^′^‐ TTA AAC TGG TTT GGG AAA ACC AAA TAT ATT‐3 ^′^ and FALr 5 ^′^‐ ACA CAA TGA ACT CAA TCA TGA CTA CCC GTC ‐3 ^′^ 205 bp; *Plasmodium vivax:* VIVf 5 ^′^‐ CGC TTC TAG CTT AAT CCA CAT AAC TGA TAC‐3 ^′^ and VIVr 5 ^′^‐ ACT TCC AAG CCG AAG CAA AGA AAG TCC TTA‐3 ^′^ 120 bp; *Plasmodium malariae:* MALf 5 ^′^‐ ATA ACA TAG TTG TAC GTT AAG AAT AAC CGC‐3 ^′^ and MALr 5 ^′^‐AAA ATT CCC ATG CAT AAA AAA TTA TAC AAA‐3 ^′^ 140 bp; and *Plasmodium ovale:* OVAf 5 ^′^‐ ATC TCT TTT GCT ATT TTT TAG TAT TGG AGA‐3 ^′^ and OVAr 5 ^′^ ‐ GGA AAA GGA CAC ATT AAT TGT ATC CTA GTG‐3 ^′^ under PCR conditions: initial denaturation at 94 ^o^C for 1 min; taking a total of 30 PCR cycles at denaturation at 94^o^C for 1 min, annealing at 58^o^C for 2 min, and elongation at 72^o^C for 5 min; final hold at 4^o^C infinitely. For quality, a known *Plasmodium*‐positive sample and a negative control sample without the DNA template were used in all reactions as positive and a negative control, respectively, to ensure the validity of the test.

#### 2.2.6. PCR for Detection of the *P. fmdr1* and the *K13 Propeller Gene*

Specific primers adopted from primers previously described by (Ariey et al. [13]) were used for amplification *Plasmodium falciparum* multidrug resistance 1 (*pfmdr1*) Fragments 186 and 184: AFR1F 5 ^′^‐AGGTTGAAAAAGAGTTGAAC‐3 ^′^ and AFR1R 5 ^′^‐ATGACACCACAAACATAAAT‐3 ^′^, AFR2F 5 ^′^‐ACAAAAAGAGTACCGCTGAAT‐3 ^′^ and AFR2R 5 ^′^‐AAACGCAAGTAATACATAAAGTC‐3 ^′^ (503 bp), *pfmdr1* Fragment 2: 1034, 1042 KFR2F 5 ^′^‐GCATTTTATAATATGCATACTG‐3 ^′^ and KFR2R 5 ^′^‐GGATTTCATAAAGTCATCAAC‐3 ^′^, KFR2F 5 ^′^‐GCATTTTATAATATGCATACTG‐3 ^′^ and KFRF2R (234 bp), *pfmdr1* Fragment 3: 1246 EFR3F 5 ^′^‐CAAACCAATCTGGATCTGCAGAAG‐3 ^′^ and EFR3R 5 ^′^‐CAATGTTGCATCTTCTCTTCC‐3 ^′^, ERFR3F 5 ^′^‐GATCTGCAGAAGATTATACTG‐3 ^′^ and EFR3R 5 ^′^‐CAATGTTGCATCTTCTCTTCC‐3 ^′^ (194 bp).

#### 2.2.7. The Amplification of 849 bp of *K13* Propeller Gene in *P. falciparum*

The primary PCR master mix was prepared in a 1.5‐mL centrifuge tube for a 25 *μ*L containing 12.5 *μ*L 2x Taq polymerase PCR ready mix, 0.5 *μ*L of 100 *μ*M of each primers, 9.5 *μ*L of nuclease‐free water and 2 *μ*L of *P. falciparum* DNA template. The PCR tubes were then labeled, and the 23 *μ*L of primary master mix was added. To each tube, 2 *μ*L of template DNA was added to the tubes and then sealed, loaded, and run in a thermocycler according to the PCR primers K133 Pf F2 5 ^′^‐GCCTTGTTGAAAGAAGCAGA‐3 ^′^ and K132 Pf R2 5 ^′^‐GCCAAGCTGCCA TTCATTTG‐3 ^′^ under the following reaction conditions: 94°C for 5 min, 94°C for 30 s, 50°C for 30 s, 68°C for 1 min (35 cycles), 68°C for 10 min, and finally held at 4°C. Second‐round PCR was performed using the second primer set (K133‐5 ^′^ GCCTTGTTGAAAGAAGCAGA‐3 ^′^ and K132‐5 ^′^GCCAAGCTGCCA TTCATTTG‐3 ^′^) and 2 *μ*L of the first‐round PCR amplicon template following the same procedure used for the first‐run PCR (Ariey et al. 2014). The second PCR product was analyzed on a 1% (*w*/*v*) agarose gel (Fisher Scientific) stained with 0.5 *μ*L of ethidium bromide. The PCR products were shipped under ambient controlled template to Ingaba (South Africa) commercial sequencing Center for Sanger sequencing. To ensure the validity of the test, a known *Plasmodium* species positive control sample and negative control sample without the DNA template were used in all reactions as a positive and negative controls, respectively.

#### 2.2.8. DNA Sequencing for *Pfmdr1* Genes and *K13* Propeller Gene

Successful PCR products showing single band in gel were then subjected to PCR purification (using FastAP alkaline phosphatase and Exonuclease I) and further processed for DNA sequencing by Sanger methods with 2x coverage (sequenced from both the forward and reverse directions) with set of specific primers for *pfmdr1* gene at Codons 86 184 AFR2F 5 ^′^‐ACAAAAAGAGTACCGCTGAAT‐3 ^′^ and AFR2R 5 ^′^AACGCAAGTAATACATAAAGTC‐3 ^′^, 1034 1042 KFR2F 5 ^′^‐GCATTTTATAATATGCATACTG‐3 ^′^ and KFRF2R 5 ^′^GGTTTAGAA GATTATTTCTGTAA‐3 ^′^, 1246, ERFR3F 5 ^′^‐ TCTGCAGAAGATTATACTG‐3 ^′^ and EFR3R 5 ^′^‐CAATGTTGCATCTTCTCTTCC‐3 ^′^ and the *K13* propeller gene k133 PfF2 5 ^′^‐GCCTTGTTGAAAGAAGCAGA‐3 ^′^ and K132 Pf R2 5 ^′^‐GCCAAGCTGCCATTCATTTG‐3 ^′^ at a reaction conditions of 94°C for 5 min, 94°C for 30 s, 50°C for 30 s, 68°C for 1 min (35 cycles), 68°C for 10 min, and final hold at 4°C. Fragments were sequenced using Nimagen, BrilliantDye Terminator Cycle Sequencing Kit V3.1, and BRD3‐100/1000, according to the manufacturer′s instructions. The labeled products were then cleaned using the ZR‐96 DNA Sequencing Clean‐up Kit (Catalogue No. D4053). The cleaned products were injected into an Applied Biosystems ABI 3500XL Genetic Analyser or Applied Biosystems ABI 3730XL Genetic Analyzer with a 50‐cm array, using POP7. Sequence chromatogram analysis was performed using molecular evolutionary genetic analysis (MEGA) Version 11.1.10 analysis software and all the sequences from a single population were aligned with the National Center for Biotechnology Information (NCBI) database. For quality control, PCR products were shipped under ambient controlled temperature to Ingaba (South Africa) commercial sequencing center for Sanger sequencing confirmation.

#### 2.2.9. Scoring of PCR Results

PCR products were stained with ethidium bromide and resolved by gel electrophoresis on 2.5% agarose gel. DNA size standards (1.5‐kb ladder) were separated alongside PCR products to allow sizing of the *pfmdr1* gene and *K13* propeller gene bands. Upon completion of gel electrophoresis, gels were placed in a gel imaging cabinet and digitally photographed under UV light. Gel images were printed and the corresponding sample lanes were scored visually for the presence of specific species. Because each nPCR was specific to only one species and a specific resistance, if present, only the corresponding amplified products appeared in the gel lane. After the images were captured, the validity of the results was checked immediately. The experiment was deemed invalid if bands were detected in the nontemplate control group.

In the case of the positive controls, a single band of the correct size was observed for each species and the resistance genes, that is, for *P. falciparum* 205 bp, *P. vivax* 120 bp, *P. malariae* 140 bp, and *P. ovale* 800 bp; for the *pfmdr1* Genes 186 and 184: 503 bp, *pfmdr1* Fragment 2: 1034, 1042 234 bp, and *pfmdr1* Fragment 3: 194 bp and 849 bp for *K13* propeller genes, respectively.

#### 2.2.10. Data Analysis

Raw data were coded and entered into EXCEL (Microsoft Excel 2016 64‐Bit Edition) and imported into IBM SPSS Version 25 (IBM SPSS Statistics 25 Grad Pack 27). To determine the prevalence of malaria parasites and gene mutations in patients at GRRH, the prevalence was calculated using the following formula:

Prevalence=number of cases of malaria or mutationsnumber of participants recruited×100%

The association between pre‐antimalarial treatment hospital visit and malaria‐negative patient after testing was determined by performing a chi‐square test.

The prevalence of *Plasmodium* parasites and gene mutations detected by the PCR method were presented using descriptive statistics, and associations were obtained using the Kruskal–Wallis test.

## 3. Results

### 3.1. Sociodemographic and Clinical Presentation of Severe Malaria in the General Participants

**Figure 1:**
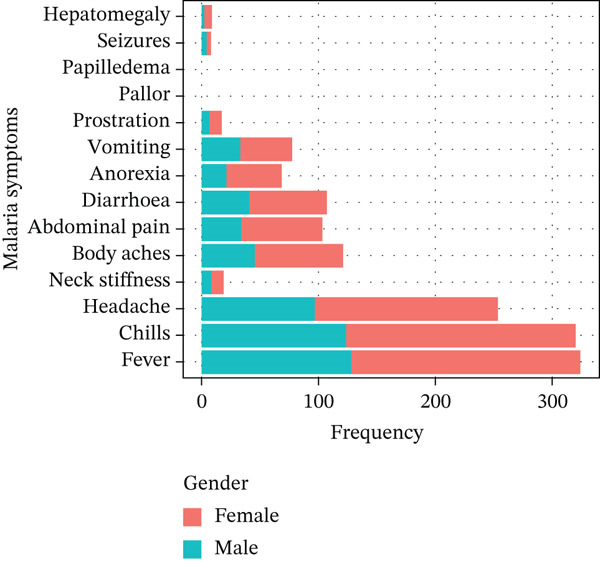
Clinical presentation of participants at GRRH. The most common clinical presentation among the general participants were fever, chills, and headache, with females, having higher frequencies than males.

Only 353 of 375 potential participants eventually signed up, yielding a response rate of 94% (*n* = 353). Females comprised 60.3% (*n* = 213) of the total participants, whereas males accounted for 39.7% (*n* = 140). The majority of participants, 91.8% (*n* = 324), were under the age of 10; 3.1% (*n* = 11) were between the ages of 11 and 20; 2.3% (*n* = 8) were between the ages of 21 and 30; 1.4% (*n* = 5) were between the ages of 31 and 40; and the remaining 1.4% (*n* = 5) were above 40. The most common clinical presentations of the participants who attended GRRH were fever (91.8%; *n* = 324), chills (90.7%; *n* = 320), headache (72.0%; *n* = 254), and body ache (34.3%; *n* = 121), respectively (Table 1). The female gender presented with most of the signs and symptoms stated than the counterparts the males (Figure 1).

**Table 1:** Malaria symptoms manifested in patients registered.

Symptoms		Responses		Percent of cases (%)
		Number	Percentage (%)	
Malaria symptoms	Fever	324	22.7	91.8
	Chills	320	22.4	90.7
	Headache	254	17.8	72.0
	Neck stiffness	18	1.3	5.1
	Body aches	121	8.5	34.3
	Abdominal pain	103	7.2	29.2
	Diarrhea	107	7.5	30.3
	Anorexia	68	4.8	19.3
	Vomiting	77	5.4	21.8
	Prostration	17	1.2	4.8
	Seizures	8	0.6	2.3
	Hepatomegaly	9	0.6	2.5
**Total**		**1426**	**100.0**	

Of the various symptoms manifested in patients reported before testing, the majority had fever at 91.8% (324), chills at 90.7% (320), headache at 72.0% (254), body aches at 34.3% (121), respectively.

### 3.2. Prevalence of *P. falciparum* Among Patients Attending GRRH by Location

The presence of *P. falciparum* in patients′ blood samples was detected using three test methods: mRDT (64.9%; *n* = 229), microscopy (63.7%; *n* = 225), and PCR (60.6%; *n* = 214). All samples that were positive by genus‐specific PCR were subjected to specific PCR to confirm the presence of different *Plasmodium* species, and the results highlight all the observed cases were due to *P. falciparum,* corresponding to a band size of 205 bp. The overall prevalence of malaria was 60.6% (*n* = 214), all of which were confirmed to be *P. falciparum,* with the highest incidence in Gulu City (24.9%; *n* = 88) and the lowest prevalence in Adjumani (1.1%; *n* = 2).

### 3.3. Symptoms, Antimalarial Treatment, and a Negative Malaria Test

**Figure 2:**
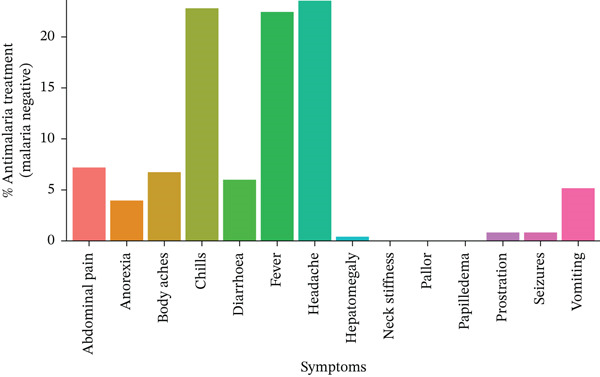
Clinical presentation in patients who reported having treatment before testing for malaria. Participants reported majorly with symptoms of fever at 21.25%, chills at 20.7%, and headache at 16.7%, respectively, and had antimalarial treatment before testing.

The highest percentage of participants reported with symptoms of fever at 21.25%, chills at 20.7%, and headache at 16.7%, respectively, and had antimalarial treatment before testing. The proportion were found negative of malaria parasites after testing, although they exhibit these symptoms relative to malarial existence in the body (Figure 2).

The antimalarial treatment was seen more in the female (60.3%; *n* = 126), with highest uptake in Gulu City (39.94%; *n* = 86) and Nwoya (15.3%; *n* = 34) (Table 2).

**Table 2:** Antimalarial treatment before the hospital visits by gender, location, and age.

		Antimalarial treatment		Percentage (%)
		No	Yes	
**Sex**	Female	87	126	60.34
	Male	57	83	39.66

Total		**144**	**209**	**100.00**

**District**	Adjumani	2	4	1.70
	Amuru	16	27	12.18
	Gulu	27	18	12.75
	Gulu City	55	86	39.94
	Nwoya	20	34	15.30
	Omoro	14	23	10.48
	Oyam	5	6	3.12
	Pader	5	11	4.53

Total		**144**	**209**	**100.00**

**Age**	< 5years	107	152	73.37
	6–10 years	24	41	18.41
	11–15 years	4	5	2.55
	16–20 years	2	0	0.57
	21–25 years	2	4	1.70
	26–30 years	1	1	0.57
	31–35years	1	2	0.85
	36–40years	0	1	0.28
	Above 40 years	3	3	1.70

Total		**144**	**209**	**100.00**

The number of patients who were negative on testing after having antimalarial treatment remains high (39.23%; *n* = 82), of the total number of patients who tested positive for malaria amidst treatment (60.77%; *n* = 127) (Table 3). The chi‐square test indicates that there was no significant association between antimalarial treatment before reporting into the hospital and malaria negative test results *X* ^2^ (1, *N* = 353) = 1, *p* = 0.517.

**Table 3:** Antimalarial treatment before reporting and malaria negative patient after testing.

Antimalarial		*P. falciparum*		Total
		Negative	Positive	
	No treatment	57	87	144
	Treatment	82	127	209
**Total**		**139**	**214**	**353**

### 3.4. Prevalence of Single‐Nucleotide Polymorphisms (SNPs) in *pfmdr1* Gene With Treatment

**Figure 3:**
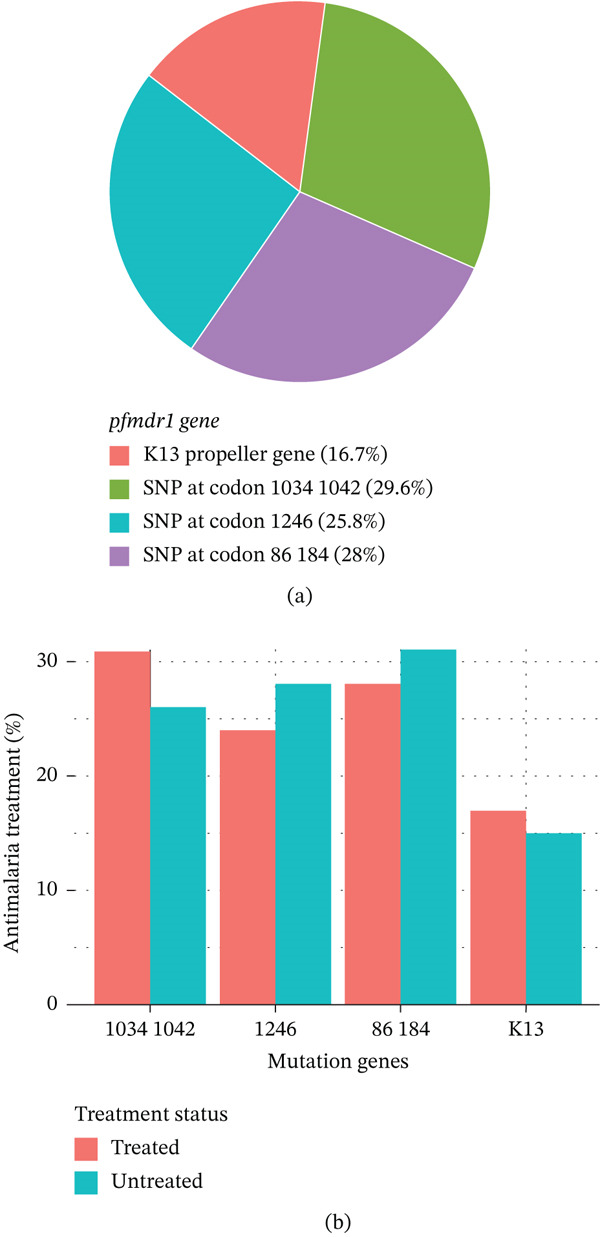
**(**a) Prevalence of gene mutations associated with *pfmdr1* and (b) mutation genes in the pfmdr1 gene occurred both in treated and untreated individuals with 1034 1042 fragments showing the highest occurrence (31%, *n* = 55).

Four SNPs within the *pfmdr1* gene associated with antimalarial drug resistance were detected: SNPs at Codons 1034 1042 (29.6%, *n* = 4), 86 184 (28%; *n* = 89), and 1246 (25.8%; *n* = 82) of the *pfmdr1* gene. Mutations within the *K13* propeller gene were observed in (16.7%; *n* = 53) of patients. Mutations at Codons 1034 1042 occurred more frequently in the treated participants (31%; *n* = 55); 86 184 and 1246 had more cases in the untreated participants (31%; *n* = 43*%*and 28*%*; *n* = 39), respectively (Figure 3). Mutations at the different codons of the *pfmdr1* gene varied with treatment status. The most frequent mutation was the SNPs at Codon 1034 1042 of the *pfmdr1* gene.

### 3.5. The Identity and Similarity of *pfmdr1* Gene Sequences With References

**Figure 4:**
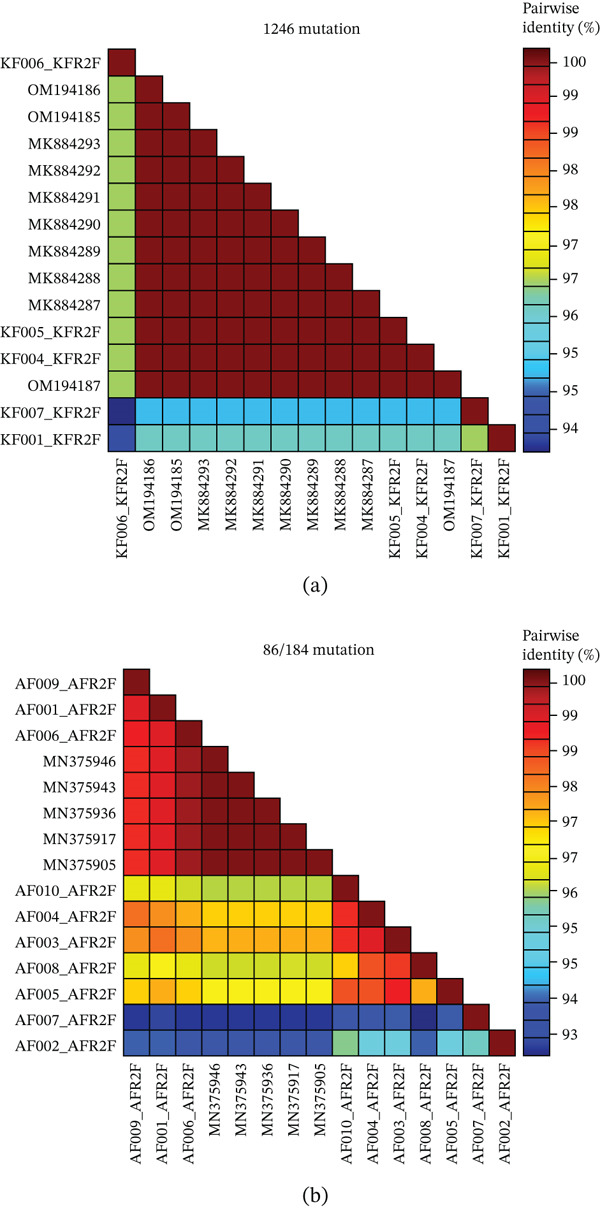
Identity matrix showed SNPs similarity of 99% at Codon 86 184, and 97% at Codon 1246 with the reference on the NCBI. The identity of the *K13* propeller genes, and SNPs at Codon 1034 1042 with the reference genes. The *K13* propeller genes and the SNPs at Codon 1034 1042 had 100% identity with the reference genes in the NCBI database (Tables 4 and 5).

The sequenced samples were aligned with the *pfmdr1* NCBI reference sequences, and all samples (NCBI Reference Samples ON903231, ON903030, MH464881, and MH464879) were found to be similar to the reference. The similarity with the NCBI were 99% at Codon 86 184, 97% at 1246 (Figure 4). The *pfmdr1* genes at Codon 1034 1042 and the *K13* propeller gene, which confers artemisinin resistance, showed 100% identity with the reference gene (Table 4). The presence of SNPs at Codons 86 184, 1034 1042, 1246, and the *K13* propeller genes indicated an association with ART resistance to malaria parasites, suggesting the potential risk of artemisinin combination therapy treatment failure in the region.

**Table 4:** The identity and similarity of *pfmdr1* gene sequences with references.

Resistance gene	Scientific name	Max score	Total score	Query 100	Percentage identity	Accession number
K 13	*P. falciparum*	1515	1515	1000%	100	828
1034 1042	*P. falciparum*	244	244	100%	100	936

**Table 5:** The nucleotide sequences on the National Center for Biotechnology Information (NCBI) database.

Bank kit	Sample ID	Accession number
BankIt2854181	K13‐010	PQ097030
BankIt2854181	K13‐009	PQ097031
BankIt2854181	K13‐008	PQ097032
BankIt2854181	K13‐006	PQ097033
BankIt2854181	K13‐003	PQ097034
BankIt2854181	K13‐001	PQ097035
BankIt2855702	AF‐001	PQ142853
BankIt2855702	AF‐002	PQ142854
BankIt2855702	AF‐003	PQ142855
BankIt2855702	AF‐004	PQ142856
BankIt2855702	AF‐005	PQ142857
BankIt2855702	AF‐006	PQ142858
BankIt2855702	AF‐008	PQ142859
BankIt2855702	AF‐009	PQ142860
BankIt2855702	AF‐010	PQ142861

### 3.6. Variation in Parasite Load by Mutation and Treatment Among Malaria Patients

**Figure 5:**
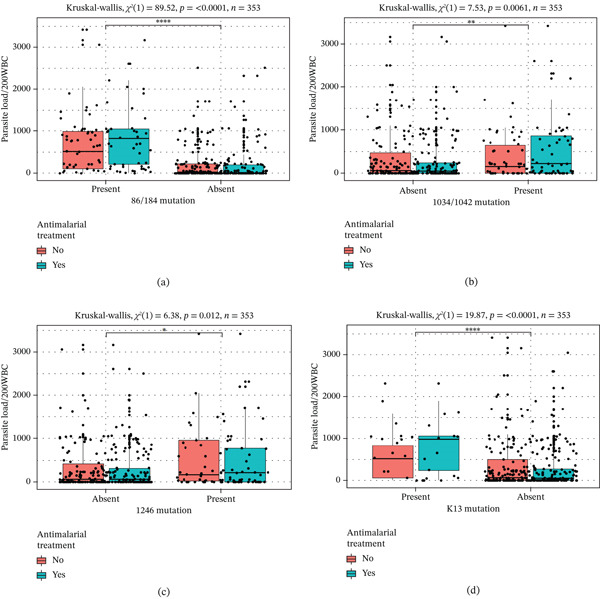
Variation in parasite load by mutation and treatment. (a) Variation in parasite load and SNPs 86 184 of the *pfmdr1* gene, (b) variation in parasite load and SNPs 1034 1042 of the *pfmdr1* gene, (c) variation in parasite load and SNPs 1246 of the *pfmdr1* gene, (d) variation in parasite load and *Kelch 13* of the pfmdr1 gene.

The median parasite load in patients with 86 184 mutations differed significantly between those who received antimalarial treatment (median = 786 parasites/200 WBC) and those who did not (median = 510 parasites/200 WBC) (Figure 5a). Patients without the 86 184 mutations generally had a lower parasite load, regardless of whether they received antimalarial treatment or not (median = 16 parasites/200 WBC) (*X* ^2^ (1) = 89.52, *p* < 0.0001, *n* = 353). Individuals with the 1034 1042 mutation showed a higher level of parasitemia, with a median of 215 parasites per 200 white blood cells (WBC) in those who received antimalarial treatment and a median of 144 parasites per 200 WBC in those who did not receive treatment (Figure 5b). Conversely, in the absence of these mutations, a lower parasite load was observed, with a median of 46 parasites per 200 WBCs in those who received antimalarial treatment and a median of 52 parasites per 200 WBCs in those who did not receive treatment. The parasite load was high in the presence of mutation 1246 in the pfmdr1 gene in patients who had received antimalarial treatment, with a median (204 parasites/200 WBCs) and 181 parasites/200 WBCs in those without treatment. In the absence of the 1246 mutation, the median parasite load was 51.5 parasites/200 WBCs in those who had received treatment and a median of 105 parasites/200 WBCs in those who had not started treatment (Figure 5c). The difference in parasitemia among patients with a 1246 mutation was significantly higher (*X* ^2^ (1) = 6.38, *p* ≤ 0.012, *n* = 353) than in patients without 1246 mutation. In the presence of *K13* propeller genes (Figure 5d), there was a higher parasite load, the median (980 parasites/200 WBCs) for patients who received antimalarial treatment and the median (507 parasites/200 WBCs) in those patients who did not receive treatment. In the absence of *K13* propeller genes, the median parasite load was (46 parasites/200 WBCs) for patients who received antimalarial treatment and median (57 parasites/200 WBCs) for patients who did not receive treatment. The difference in parasitemia among patients with a mutation in the *K13* propeller genes was significantly (*X* ^2^ (1) = 19.87, *p* ≤ 0.0001, *n* = 353) higher than that in patients without mutations in the *K13* propeller genes.

## 4. Discussion

Malaria is the most prevalent tropical disease in the world today, and in SSA, it is ranked among the most frequent causes of morbidity and mortality. Uganda is among the 29 malaria endemic countries that contributed to 95% of global malaria cases in 2019, especially among children [14], and is often the leading identifiable cause of death [15]. Based on the above, countries within SSA set up strategies to eradicate malaria and one of the approaches was to identify resistant genes [16]. Therefore, this study focused on identifying multidrug resistance genes conferring resistance to antimalarial drugs.

There was a positive response rate of 94% in this study, and the sociodemographic and clinical characteristics of participants with suspected cases of malaria in the present study showed the female gender of 60.3%, accounting for the highest number of respondents than the male counterparts. The most common clinical presentations among the participants in the study were fever (91.8%), chills (90.7%), and headaches (72.0%). These observations are consistent with other studies that suggest that fever with chills, and rigor is the most common mode of presentation of severe malaria in SSA [17].

Molecular screening using PCR assays in this study revealed that more than half of the samples 60.6% were positive for *P. falciparum,* with more females being affected than males, which is in agreement with the report by [18]. The significantly high prevalence of the high burden of malaria infection in this study may have coincided with the limited control options and availability of resources in the study areas. This may also be a result of the timing of sample collection due to the malaria transmission season. The highest cases of *P. falciparum* infection were registered in Gulu City. This was because GRRH is located within Gulu City; therefore, there was easy accessibility to the hospital and free treatment to the community within and near Gulu City. As a referral hospital, all critical cases were referred for better management, resulting in a high prevalence. The high prevalence of *P. falciparum* in Gulu City may therefore be attributed to the permeating physical fear of inadequate malaria commodities, which is noteworthy considering the anticipated doubling of malaria deaths in SSA in 2020, as reported by [19]. There was also the possibility of an upsurge in the number of resistant strains due to incorrect treatment or delayed treatment due to misdiagnosis of malaria cases. This was in line with a previous study in Northern Uganda, an area of high malaria transmission, which showed a likelihood of growing resistance to ACTs [20].

The study found that participants have self‐diagnosed malaria basing on the clinical symptoms mainly fever, chills, and headache, and they had reported having had antimalarial treatment before hospital visit, and they turned out to be negative on testing for malaria; however, there was no significant association between antimalarial treatment before reporting into the hospital and malaria negative test results *p* = 0.517. This was because most of the participants assumed that the cause of fever is actually malaria, and that malaria was not serious illness; hence, they normally do a self‐medication however this treatment does not resolve symptoms like fever, chills, and headache, thus prompting them to have a hospital visit and also with a negative result hence negative malaria test results and antimalarial resistance. This was in line with the study done by [21] where antimalarial drug failures have been reported anecdotally in Nigeria, and malarial self‐treatment practices could be a contributing factor. The exposure to antimalarial by the participants before the hospital visits was high as seen in the female, with the highest uptake in Gulu City and among those aged < 5 years. This could have been mainly due to ease of access through the over the counters, pharmacies, and or medications from previous illnesses. This was in concordance with the finding in the study by [22] where the prehospital exposure to antimalarial in the study setting was high, and the determining factors to prehospital antimalarial exposures include the duration of fever, level of the nearest health facility, distance from the nearest health facility, and caregivers′ age.

Samples that were positive for *P. falciparum* were evaluated for SNPs and SNPs at codons 86 184, 1034 1042, and 1246 in the *pfmdr1* and *K13* propeller genes. The SNP 1034 1042 was the most prevalent, with the highest occurrence in Gulu City. The study also found that mutations at codons 1034 1042 occurred more frequently in the treated participants (31%; *n* = 55), and 86 184 and 1246 had more cases in the untreated participants (31%; *n* = 43 and 28%; *n* = 39), respectively. The finding in this study was in agreement with the report that mutations at the different codons of the *pfmdr 1* gene varied with treatment status and, that resistance to antimalarial drugs is mainly associated with SNPs that occur at altered genetic loci of resistant genes [23]. In Gulu City, the occurrence of four different mutations, SNPs 86 184, 1034 1042, 1246, and *K13,* were dominant throughout the district. SNPs at specific codons in both the *pfmdr1* and *K13* propeller genes can have significant implications for antimalarial drug resistance. These SNPs influence the parasite response to different antimalarials and play a crucial role in treatment outcomes and the spread of drug‐resistant malaria. It is also important to consider that the effects of these mutations can vary depending on the parasite strain and the presence of other ACTs as a frontline treatment for uncomplicated malaria caused by *P. falciparum*, the most severe form of malaria.

This study also revealed a 16.7% existence of mutation in the *K13* propeller gene among participants with *P. falciparum* infection, which was higher than the findings of [4], who reported a 15.8% *K13* mutation rate. Numerous mutational markers associated with artemisinin resistance have been identified in in vitro drug adaptation studies [6]. Mutations in *K13* gene are primarily associated with resistance to artemisinin and its derivatives, which are crucial components of ACTs. This was identified to be definitively connected with artemisinin resistance as reported by [5]. These SNPs in the *K13* gene were also reported in other African countries [24]. The occurrence of the *K13* propeller genes in this study confirmed resistance to artemisinin in the study area.

On evaluating the variation in parasite load and mutation among the study participants, the findings revealed that the difference in median parasite load in patients with Mutations 86 184, 1034 1042, and *K13* propeller genes did not significantly vary among the patients who received antimalarial treatment *p* < 0.0001, *p* = 0.0061, and *p* = 0.012, respectively. Previous studies have reported that mutations are generally associated with fitness costs but are selected because they enhance the survival of the parasite in the presence of the drug; however, upon removal of the pressure, the mutation no longer has a selective advantage [8]. This current study discovered that amidst treatment, there was high parasitemia in the presence of almost all the confirmed mutations, which is an indication that mutations are the gear to malaria resistance, which might have resulted from the high misuse of drugs, underdosing, overdosing, or constant exposure of parasites to drugs. The finding in this study agrees with the findings of a previous study that showed that the presence of these mutant genes could be a result of the high indiscriminate use of drugs (drug abuse) for the treatment of malaria by individuals. This could also be a result of the long‐time use of artemisinin as an antimalarial drug. Constant exposure of the parasite to drugs can lead to the development of these resistance genes.

The findings in this study also showed that in the presence of *K13* propeller genes, there was a much higher parasite load, median for patients who received antimalarial treatment *p* ≤ 0.0001, indicating the importance of the *K13* propeller gene in antimalarial resistance. In a previous study of the *pfk13*‐propeller gene, SNPs arising because of mutations have been considered to confer resistance to ACTs [25]. This has been supported by previous reports indicating that artemisinin resistance is caused by mutations in theK13 protein encoded by *PF3D7_1343700* on *P. falciparum* Chromosome 13 (*K13*) [3]. The current study carried out in Northern Uganda (GRRH) confirmed the presence of *K13* mutation genes that confer artemisinin resistance, which is in concordance with a previous study carried out in Northern Uganda, which is a high malaria transmission area and indicates the possibility of increasing resistance to ACTs [20].

## 5. Conclusion

Malaria prevalence was high among people attending GRRH, with the highest cases registered in females and in Gulu City. The treatment with antimalarial before hospital visits in the study was high, driven by the ease of access through drug shops, pharmacies, and clinics and the use of remnant medications from previous illnesses. This pretreatment was mainly due to the duration of fever, level of the nearest distance from the nearest health facility, and caregivers′ age. There was also a high prevalence of SNPs and *K13* in *P. falciparum* parasite‐positive samples. The presence of mutations was also associated with high parasitemia, despite treatment. However, with the above, there could also be other resistance genes that may confer resistance to the antimalarial that this study could not have exhausted, considering the increasing rate of malaria infection.

## Author Contributions

F.P.A.: conceptualization, proposal development, methodology, data collection, and manuscript writing. G.W.: acted as technical advisor, field supervisor for the study data analysis, and manuscript writer. H.A.: proposal development, methodology, supervision, and manuscript writing. L.O.: proposal development, methodology, supervision, data analysis, and manuscript writing. R.O.: supervision and manuscript writing. S.O.: acted as technical advisor on methodology and data collection, analyzing data, and manuscript writing and refinement. Q.O.: acted as technical advisor on sample collection and field supervisor for the study at the data collection site. R.E.: proposal development, methodology, data collection, data analysis, and manuscript writing.

## Funding

No funding was received for this manuscript.

## Ethics Statement

Ethical approval and consent waivers for this study were obtained from Gulu University Research and Ethic Committee (GUREC‐2022‐262), Gulu Regional Referral Hospital Research and Ethics Committee (GRRHREC‐ ADM/2022‐07/040), and Uganda National Council of Science and Technology (NS393ES). Permission to conduct the study in the hospital was obtained from the medical superintendent and head of department laboratory services of Gulu Regional Referral Hospital. Concealment was ensured by keeping the collected data secure under key and lock. This study conformed to the ethical standards in Uganda.

## Consent

Institutional approval for the publication of this manuscript was obtained from the Gulu University Multifunctional Research Laboratories and Department of Biology, Faculty of Science, Gulu University, Gulu, Uganda.

## Conflicts of Interest

The authors declare no conflicts of interest.

## Data Availability

The study data are available upon request from the corresponding author. The nucleotide sequences have been submitted to the National Center for Biotechnology Information (NCBI) database and were assigned GenBank accession numbers for your nucleotide sequences as seen in Table 5.
